# Evaluation of bacterial hosts for conversion of lignin-derived *p*-coumaric acid to 4-vinylphenol

**DOI:** 10.1186/s12934-021-01670-8

**Published:** 2021-09-15

**Authors:** Alberto Rodriguez, Jamie A. Meadows, Ning Sun, Blake A. Simmons, John M. Gladden

**Affiliations:** 1 Joint BioEnergy Institute, 5885 Hollis St, Emeryville, CA 94608 USA; 2 Sandia National Laboratories, 7011 East Ave, Livermore, CA 94551 USA; 3 Lawrence Berkeley National Laboratory, 1 Cyclotron Rd, Berkeley, CA 94720 USA

**Keywords:** 4-vinylphenol, Coumaric acid, 4-hydroxystyrene, Microbial conversion, Lignin

## Abstract

Hydroxycinnamic acids such as *p*-coumaric acid (CA) are chemically linked to lignin in grassy biomass with fairly labile ester bonds and therefore represent a straightforward opportunity to extract and valorize lignin components. In this work, we investigated the enzymatic conversion of CA extracted from lignocellulose to 4-vinylphenol (4VP) by expressing a microbial phenolic acid decarboxylase in *Corynebacterium glutamicum*, *Escherichia coli,* and *Bacillus subtilis*. The performance of the recombinant strains was evaluated in response to the substrate concentration in rich medium or a lignin liquor and the addition of an organic overlay to perform a continuous product extraction in batch cultures. We found that using undecanol as an overlay enhanced the 4VP titers under high substrate concentrations, while extracting > 97% of the product from the aqueous phase. *C. glutamicum* showed the highest tolerance to CA and resulted in the accumulation of up to 187 g/L of 4VP from pure CA in the overlay with a 90% yield when using rich media, or 17 g/L of 4VP with a 73% yield from CA extracted from lignin. These results indicate that *C. glutamicum* is a suitable host for the high-level production of 4VP and that further bioprocess engineering strategies should be explored to optimize the production, extraction, and purification of 4VP from lignin with this organism.

**Supplementary Information:**

The online version contains supplementary material available at 10.1186/s12934-021-01670-8.

## Background

The discovery of sustainable and affordable routes for the production of commodity and specialty biochemicals from renewable sources is essential to reduce the global demand for petroleum and other fossil materials. Lignocellulosic biomass comprises one of the largest reservoirs of renewable carbon in the planet and represents an attractive source of chemical building blocks that can be refined and upgraded into biofuels and bioproducts ([14, 15]). Among the main constituents of lignocellulosic biomass, cellulose and hemicellulose are polymeric carbohydrates that can be deconstructed to monomeric sugars and converted to biofuels and other valuable bioproducts [4]. The third main component is lignin, an aromatic heteropolymer with high chemical complexity that makes it more resistant to deconstruction and conversion [30]. Considering that this complexity also opens the possibility to valorize a wide array of aromatic compounds, efforts to depolymerize lignin and chemically or biologically upgrade lignin monomers have steadily increased over the last decade [2, 23, 29]. For example, hydroxycinnamic acids such as *p*-coumaric (CA) and ferulic (FA) acids are precursors of lignin biosynthesis in plants and are chemically linked to lignin grassy biomass at considerable levels (5–15% wt.) [20, 26]. These types of compounds could represent a viable option for valorization of aromatic compounds derived from lignocellulosic feedstocks because they can be extracted through mild thermochemical treatments, are fully metabolized by certain fungal and bacterial species, and their relative amounts could be increased through bioengineering of crops [14, 15, 21, 24]. In particular, CA and FA can be decarboxylated to their vinylphenol derivatives, which hold a high market value due to their applications as biopolymer precursors, perfumes, and food additives.

Several groups have explored the feasibility of using enzymatic approaches to convert CA to 4-vinylphenol (4VP) or FA to 4-vinylguaiacol (4VG) through a single reaction catalyzed by a phenolic acid decarboxylase (PAD) enzyme [5, 8, 10, 16, 16, 17, 17]. Over 100 bacterial species are predicted to contain genes coding for PAD enzymes but the best characterized enzymes to date belong to members of the *Bacillus* and *Lactobacillus* genera [18]. In addition to using organisms with the natural ability to decarboxylate CA or FA, several PAD enzymes have been heterologously expressed in *E. coli* and either purified for in vitro reactions or the recombinant strains used in whole-cell conversion methods [1, 10, 25]. However, 4VP has proven to be significantly more toxic to *E. coli* than CA and there are reports of product inhibition in PAD enzymes [12]. Some strategies that have been employed to mitigate this problem include the use of biphasic systems with an organic overlay, extractive fermentation, and fed-batch approaches. In particular, hexane and octanol are two organic solvents that have proven to be effective at extracting 4VP from the aqueous phase but are also known to cause significant toxicity to *E. coli* and inhibition of PAD enzyme activity [10, 16, 17, 25].

Although some organisms, such as *Pseudomonas putida* and *Streptomyces mobaraense,* have been used for the production of 4VP from glucose [7, 27] or grass lignin feedstocks [28], the production of 4VP from CA has been attempted primarily in *E. coli*. Here we explored whether other bacterial species could be more tolerant to higher, more industrially relevant concentrations of substrate and product and to the presence of an organic overlay for product extraction. To do so, the PAD gene from *Bacillus amyloliquefaciens* was heterologously expressed in *Corynebacterium glutamicum*, *Bacillus subtilis,* and *E. coli*, and their 4VP production capabilities were determined using high-substrate batch cultivations. In an attempt to reduce the toxicity of the overlay, the performance of longer chain fatty alcohols, such as decanol and undecanol, was compared to the more commonly used overlay, octanol. Finally, liquors containing CA extracted from a lignin-rich fraction of lignocellulose were generated through an alkaline treatment and the strains were tested for 4VP production. The 4VP yields and titers obtained under these conditions were compared to those obtained from pure CA in rich medium.

## Results and discussion

### Initial characterization of strains

Three bacterial species were selected in this study for overexpression of a PAD enzyme: *E. coli* (Ec), *B. subtilis* (Bs) and *C. glutamicum* (Cg). Their ability to convert CA to 4VP was evaluated and compared to the species carrying the native gene, *B. amyloliquefaciens* (Ba). These organisms were chosen because they are common protein expression hosts with multiple engineering tools available and do not consume CA or 4VP, with the exception of Cg that is capable of assimilating CA. The metabolic pathway for assimilation of CA and other phenylpropanoids in *C. glutamicum* has been described in detail by Kallscheuer et al., and interested readers are encouraged to consult that reference [11]. In an attempt to maximize CA conversion to 4VP in Cg, a clean deletion of *phdA* (coding for an acyl:CoA ligase required for CA degradation) was made to generate a strain unable to metabolize CA (Cg phdA-). It is also worth noting that wild-type Bs harbors a gene coding for a PAD enzyme in its genome; however, a plasmid-based expression is expected to have a positive effect on product formation rates and titers. The PAD from Ba was selected in this work to be overexpressed in all organisms based on a previous study that identified it as having high specific activity [10]. However, the purpose of this work was not to study the performance of this particular enzyme but rather to determine if organisms such as Cg and Bs can be efficient 4VP producers.

The Ec, Cg and Cg phdA- strains were transformed with plasmids containing codon-optimized versions of the Ba PAD under the control of the strong IPTG-inducible promoter Ptac, called pJAM88 and pJAM89 (Table 1). These strains were named Ec PAD, Cg PAD and Cg phdA- PAD. Bs was transformed with a similar IPTG-inducible plasmid pJAM90 containing a codon-optimized version of the Ba PAD gene controlled by the strong promoter Pgrac [19], resulting in strain Bs PAD.

**Table 1:** Strains and plasmids used in this work. The materials generated in this work are stored at the Joint BioEnergy Institute (JBEI) and can be accessed at https://public-registry.jbei.org with the registry ID numbers provided

Strain name	Genotype or description	Source	JBEI Registry ID
Cg	*Corynebacterium glutamicum* ATCC 13,032	ATCC	
Cg *ΔphdA*	*Corynebacterium glutamicum* Δ*phdA*	This study	JPUB_018349
Cg PAD	*Corynebacterium glutamicum* wild type with pJAM89	This study	JPUB_018350
Cg *ΔphdA* PAD	*Corynebacterium glutamicum* Δ*phdA* with pJAM89	This study	JPUB_018352
Bs	*Bacillus subtilis* RIK1285 (strain 168 derivative)	TakaraBio	
Bs PAD	*Bacillus subtilis* RIK1285 with pJAM90	This study	JPUB_018354
Ba	*Bacillus amyloliquefaciens* ATCC 23,350	ATCC	
Ec	*Escherichia coli* DH5α	Zymo Research	
Ec PAD	*Escherichia coli* DH5α with pJAM88	This study	JPUB_018346

Plasmid name	Description	Source	JBEI Registry ID
pK18mobsacB	suicide vector	ATCC	
pZ8-Ptac	IPTG-inducible strong promoter expression vector	Addgene [6]	
pHT08	IPTG-inducible strong promoter expression vector	MoBiTec	
pJAM88	pZ8-Ptac with PAD codon-optimized for *E. coli*	This study	JPUB_018347
pJAM89	pZ8-Ptac with PAD codon-optimized for *C. glutamicum*	This study	JPUB_018351
pJAM90	pHT-08 with PAD codon-optimized for *B. subtilis*	This study	JPUB_018355

To test whether the PAD strains were able to convert CA to 4VP and evaluate their tolerance to different concentrations of substrate and product, growth experiments were performed in tryptic soy broth supplemented with 0.25 or 0.5 g/L of 4VP, or 1, 5 or 10 g/L of CA. This rich medium was used to provide all the organisms with glucose and amino acids for growth and cell maintenance and allow for continuous PAD protein production during the 4VP production process. The bacterial cell density was measured at the time of inoculation and after 24 h of incubation, and PAD expression was induced at the beginning of the cultivations.

**Fig. 1:**
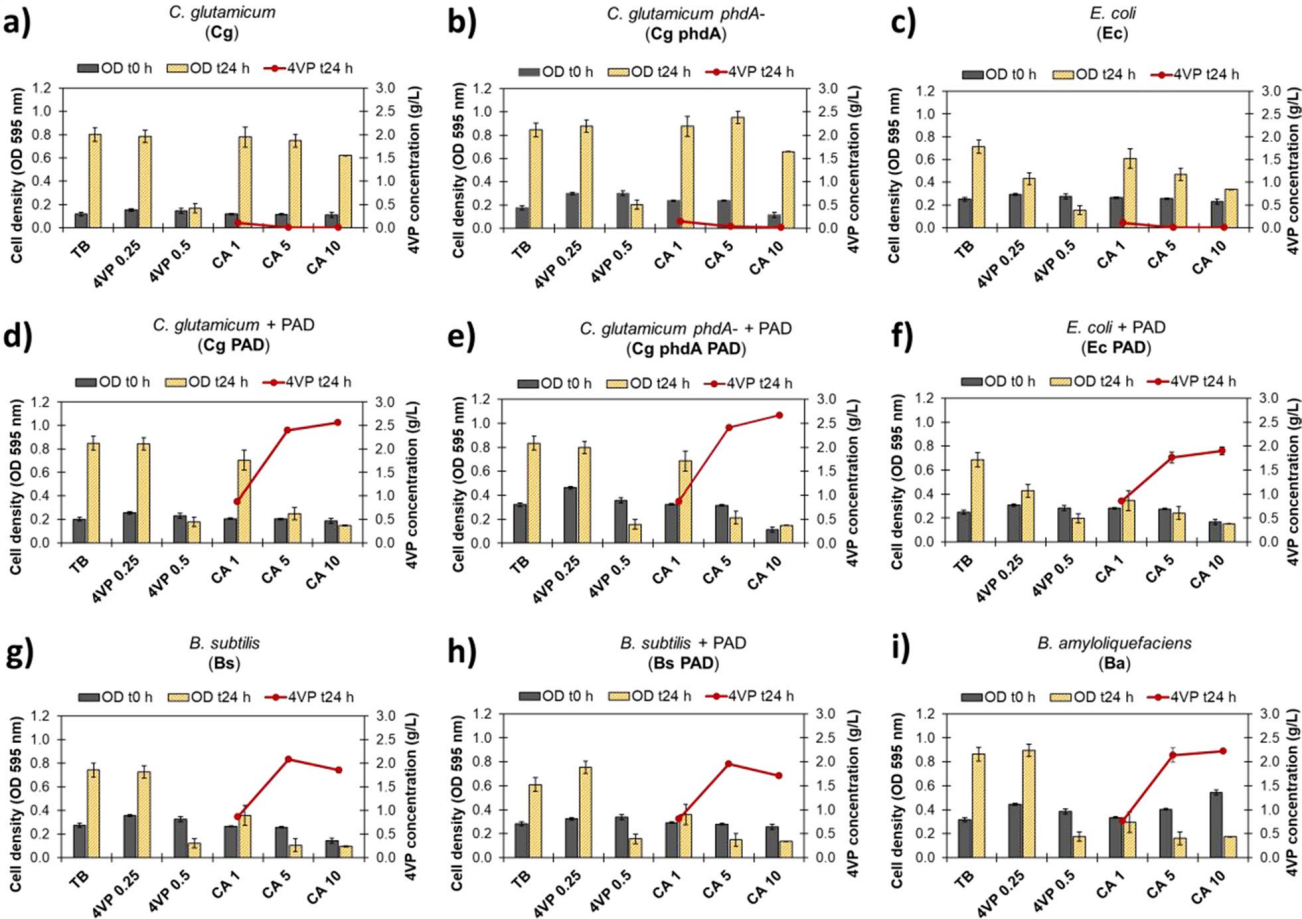
Cell density values measured during cultivation of the 9 strains in tryptic soy broth supplemented with 4VP or CA at different concentrations. Numbers in the horizontal axis indicate initial concentrations of 4VP or CA in grams per liter. TB refers to tryptic soy broth without any supplements. The 4VP concentrations were measured after 24 h of cultivation. The strains used are *C. glutamicum* (**a**),* C. glutamicum phdA-* (**b**),* E. coli* (**c**),* C. glutamicum* + PAD (**d**),* C. glutamicum phdA-* + PAD (**e**),* E. coli* + PAD (**f**),* B. subtilis* (**g**),* B. subtilis* + PAD (**h**), and* B. amyloiquefaciens* (**i**)

The results show that 4VP is very toxic to all tested organisms, although Ec appeared to be the most sensitive, displaying growth inhibition at concentrations as low as 0.25 g/L (Fig. 1c, f). This agrees with previously reported 4VP toxicity values for other *E. coli* strains [12]. Cg and Cg phdA- strains showed the highest tolerance to CA by being able to grow in the presence of up to 10 g/L of this compound (Fig. 1a, b), while all *Bacillus* species were unable to grow in media containing more than 1 g/L of CA (Fig. 1g–i).

Notably, the transformed Cg, Cg phdA-, and Ec cells exhibited increased sensitivity to CA compared to the non-transformed variants, suggesting that 4VP formation (and indirectly the initial CA concentration) is causing growth inhibition as a result of PAD expression. We confirmed that 4VP was produced by all strains that harbor a PAD in media containing CA (Fig. 1). As expected, Bs and Ba can naturally produce 4VP but not the untransformed Cg or Ec strains. Interestingly, Bs and Ba wild-type strains produced similar 4VP amounts as the transformed strains in this experiment, even when the cell density in the cultures did not increase. Product concentrations reached a plateau around 2.5 g/L despite increasing the initial CA concentration from 5 to 10 g/L. These observations indicate that even a small amount of PAD enzyme (cell densities as low as 0.1 OD) may be enough to reach inhibitory product concentrations and that 4VP toxicity (and not substrate availability) is likely limiting the product titers under these conditions.

### Use of an organic overlay and high coumarate concentrations

Previous reports have found that the microbial production of 4VP can be enhanced by using a biphasic cultivation system. One example is the extraction of 4VP from the aqueous phase with octanol in a continuous flow-bed reactor containing immobilized *E. coli* cells [10]. Although the partition coefficient for 4VP in a mixture of octanol and water is predicted to be high towards octanol, this solvent is also known to cause significant toxicity to microbes [3]. Therefore, we compared the performance of octanol to other mid-chain fatty alcohols with slightly longer alkane chains, decanol and undecanol, when added as overlays to the cultivation media (20% of the aqueous phase volume). To obtain high product concentrations and identify any limitations to the amount of 4VP that can be extracted with the overlays, the four recombinant strains were incubated in the presence of 25 or 75 g/L of CA.

**Fig. 2.**
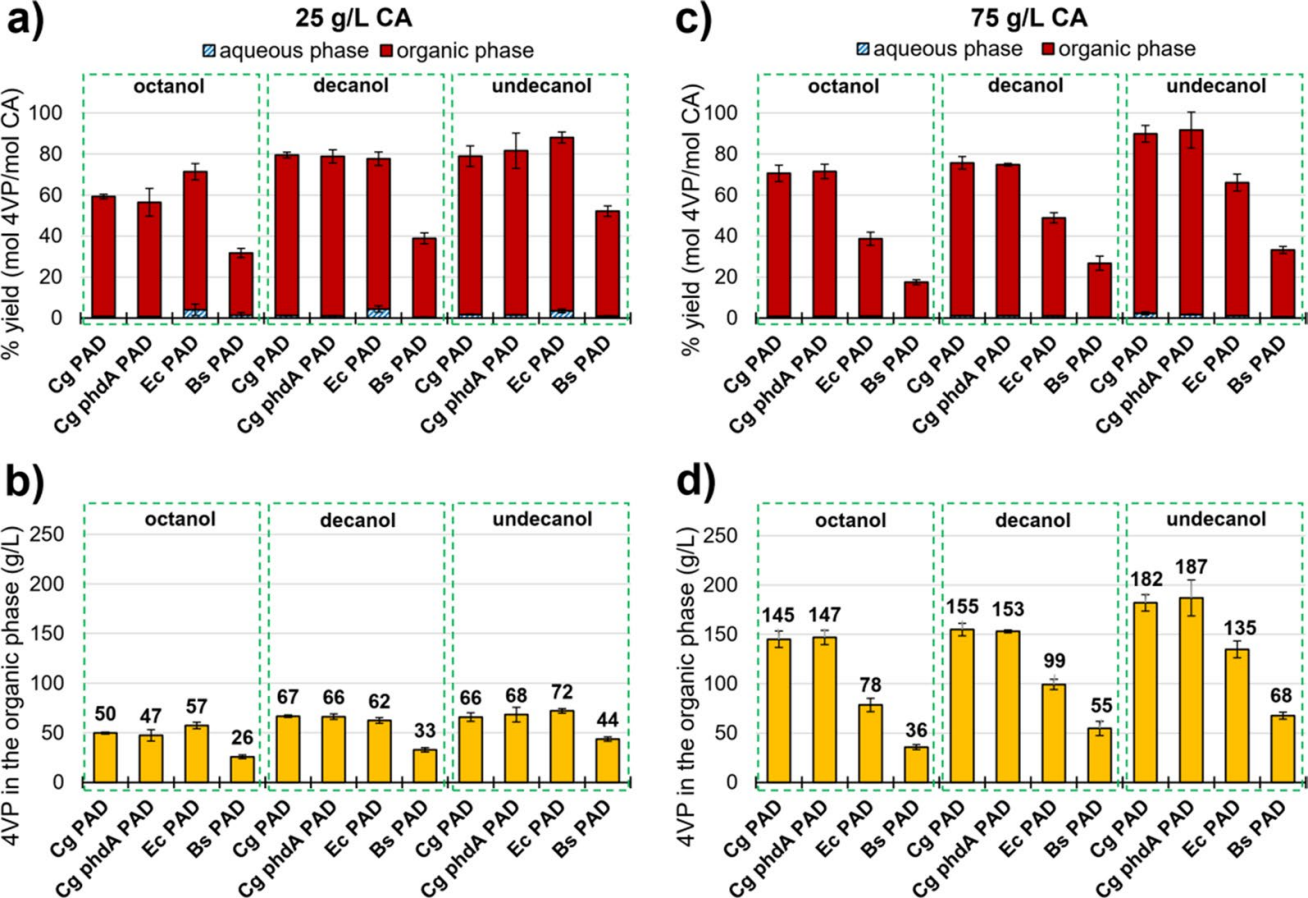
4VP yields (**a**, **c**) and concentrations (**b**, **d**) obtained in experiments using tryptic soy broth containing 25 g/L (**a**, **b**) or 75 g/L (**c**, **d**) of CA. Experiments were inoculated at an OD of 4 and yields were calculated based on the concentrations of 4VP in the aqueous and organic phase relative to the initial CA after 48 h of cultivation

We observed differences in the 4VP yields and titers obtained with each overlay and organism. For example, Bs showed lower conversion yields and product titers than Cg or Ec (Fig. 2). We found that undecanol performs as well or better than octanol and decanol in terms of 4VP yields and titers, and this effect appears stronger when using a higher initial CA concentration. Although the titers and yields obtained with Cg and Ec in the presence of 25 g/L of CA were similar, Cg performed better in 75 g/L of CA, reaching a concentration of 4VP of 187 g/L in the undecanol overlay with a 90% conversion yield. This could be a consequence of the higher tolerance of this microorganism to CA, as discussed previously. Interestingly, no difference in 4VP production was found between Cg and Cg phdA- strains, which suggests that the CA assimilation pathway is not particularly active in the wild-type strain under these conditions.

Remarkably, over 97% of the 4VP produced was found in the organic phase, while more than 90% of the CA that was not consumed was found in the aqueous phase. This indicates that the three alcohols are able to efficiently extract and concentrate 4VP from the aqueous phase, while the substrate remains accessible to the cells. The use of an overlay markedly increased the production of 4VP when compared to monophasic cultures (Fig. 1). Undecanol was selected for further experiments based on the results shown in Fig. 2c, d and because it is the mid-chain fatty alcohol with the longest alkane chain that is liquid at room temperature, which could facilitate product recovery from the fermentation broth. It is important to consider that, besides helping cells to produce 4VP for a longer period of time, the inclusion of an overlay also helps to concentrate the product in a smaller volume and may be beneficial for a subsequent purification step.

### 4VP production from lignin-extracted CA

**Fig. 3:**
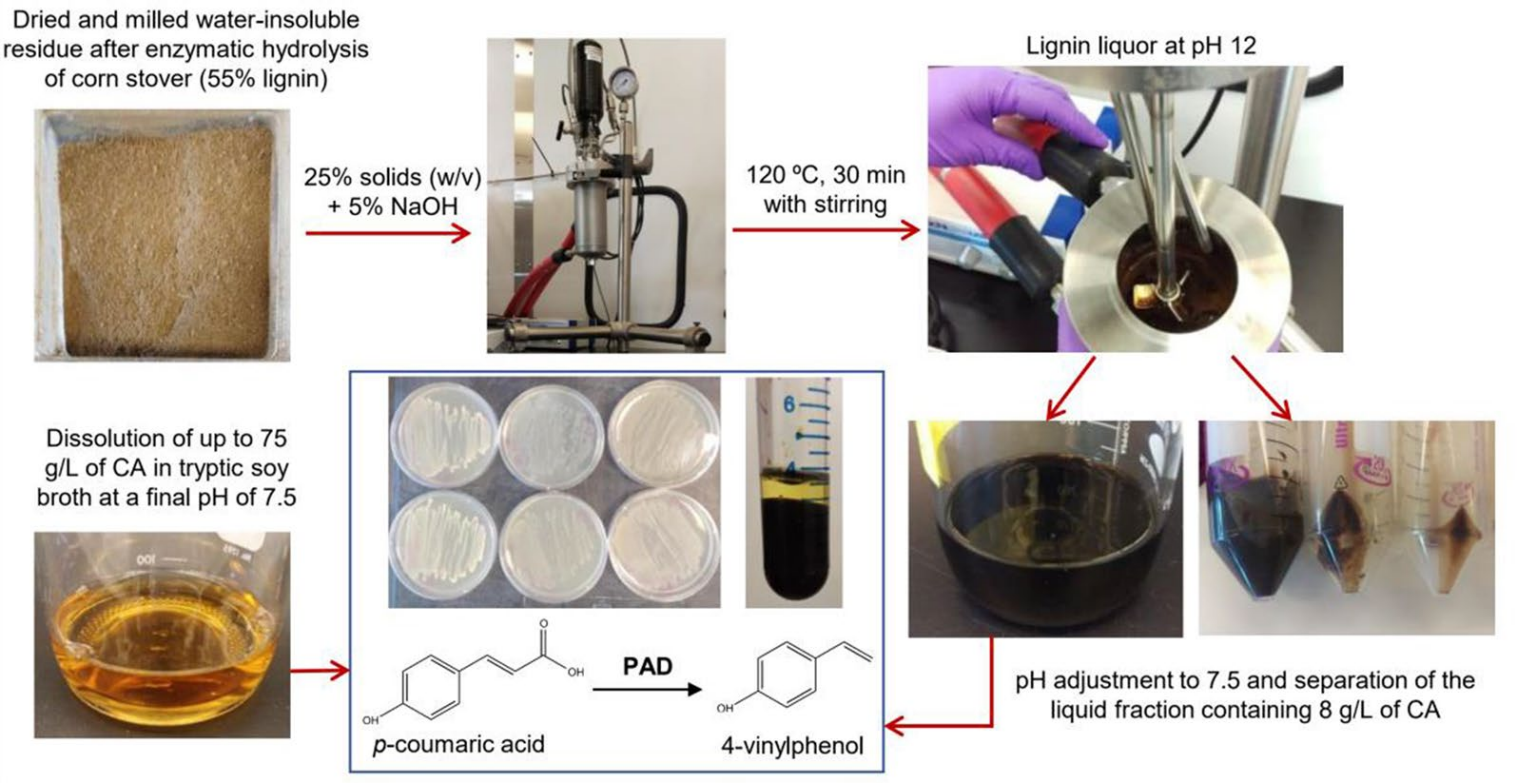
Scheme of the microbial production of 4-vinylphenol (4VP) from *p*-coumaric acid (CA) in rich media or lignin-derived CA. PAD = phenolic acid decarboxylase

To explore a renewable source of 4VP production, the strains generated in this work were also tested using CA derived from lignocellulose. The lignin-rich substrate used in this study was derived from a dilute acid pretreatment and enzymatic saccharification of corn stover and is water insoluble. This substrate represents a typical solid waste stream that might be generated in a lignocellulosic biorefinery. To extract CA, the lignin-rich solid residue must be first solubilized under conditions that both promote the release of CA and generate a biocompatible liquor (i.e. having a pH and salt concentrations within the physiological range of the microbes and low amounts of toxic phenolic compounds). Previous reports indicate that CA yields close to 10 wt% of the total lignin-rich solids content can be obtained with a base-catalyzed depolymerization method using corn stover lignin [24]. Here, we applied a similar approach to prepare lignin liquors from the water-insoluble residue of corn stover (containing 55 wt% lignin), obtained after acid pretreatment and saccharification reactions in a pilot-scale process. A relatively high solids load of 25% (w/v) was used in the depolymerization reaction to maximize the CA concentration in solution (Fig. 3). The CA-rich liquor was pH-adjusted to 7.5 and a CA concentration of 8 g/L was measured. CA was the main compound detected in HPLC–UV chromatograms obtained from this material (Additional file 1).

The lignocellulose-derived CA was used to cultivate the 4VP producing strains. The cultivation setup used in the experiment shown in Fig. 2 included the use of multi-well plates and high initial cell densities (as detailed in Methods). This was done to facilitate the screening of all combinations of strains, solvents, and substrate amounts, while minimizing the toxicity imposed by the high CA concentrations. However, this culture system is not amenable for repeated sampling of aqueous and organic phases to quantify metabolites or for monitoring cell growth in the presence of overlays and dark lignin liquors. Therefore, the incubations in CA-rich liquors were performed in culture tubes in presence of an undecanol overlay, and the OD, CA and 4VP concentrations were measured at 0, 12 and 24 h after inoculation. Considering that an organism able to produce 4VP at a high concentration and yield from lignin should not consume CA as a carbon source and may not necessarily be able to grow on lignin, high initial cell densities (OD ~ 3.5) were also used in this experiment.

**Fig. 4.**
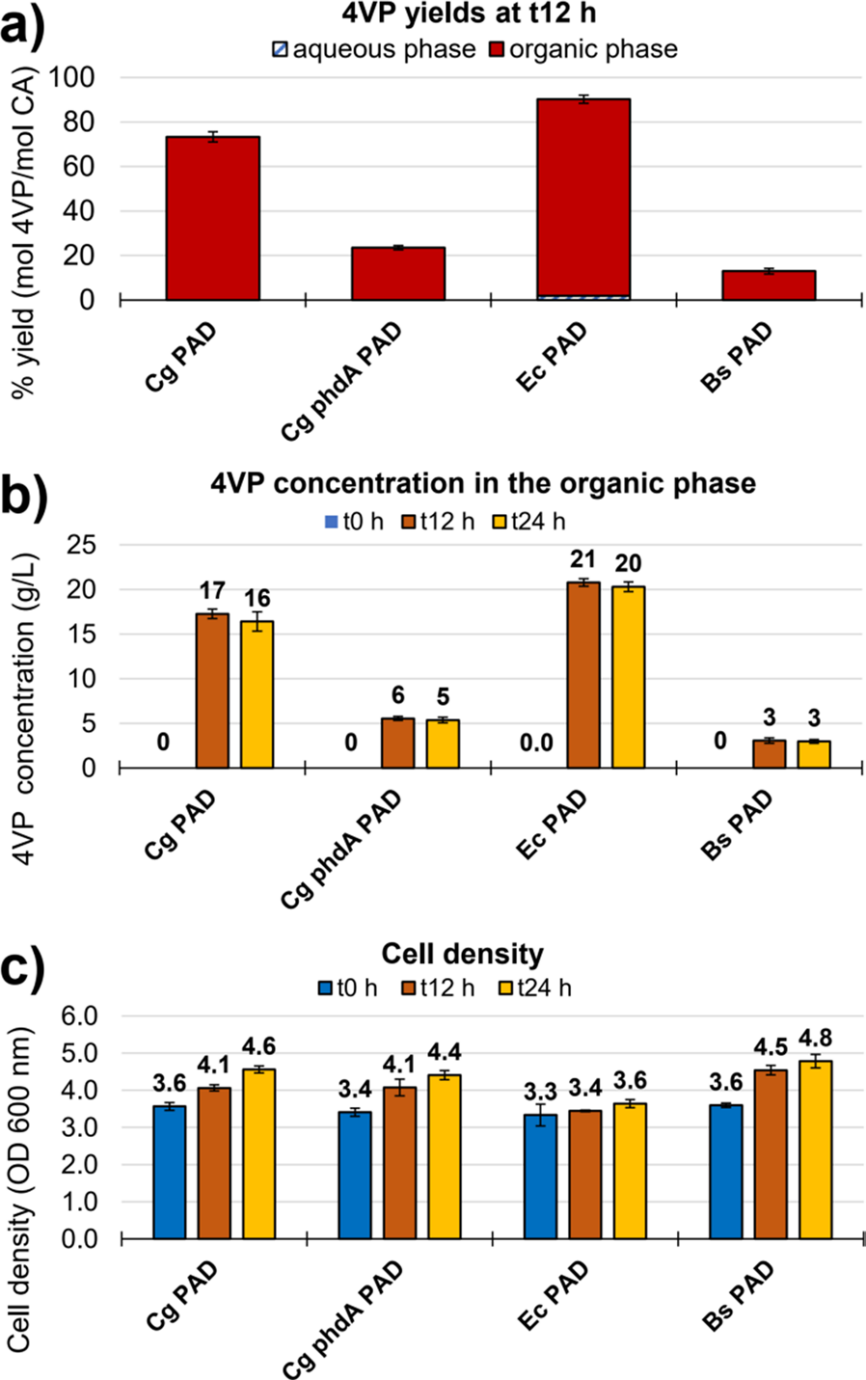
4VP yields (**a**), concentrations (**b**), and cell densities (**c**) obtained when cultivating the producing strains in lignin liquors. Yields were calculated based on the concentrations of 4VP in the aqueous and organic phase relative to the initial CA after 48 h of cultivation

Maximum product concentrations were obtained within 12 h in all cases (Fig. 4). The Ec PAD strain showed the highest product concentration, reaching 21 g/L of 4VP with an 88% yield, followed by strain Cg PAD that produced 17 g/L with a 73% yield (Fig. 4). The Bs PAD strain was once again the lowest 4VP producer, accumulating only 3 g/L of 4VP. Interestingly, the Cg phdA- PAD strain resulted in low production values under these conditions (6 g/L of 4VP), suggesting that the absence of the acyl:CoA ligase could have adverse consequences for Cg when cultured in complex lignocellulose-derived media. Overall, the cell densities increased only slightly over the course of the cultivation but no correlation to the 4VP production values was found.

It is clear that the use of concentrated lignin liquors poses additional challenges to the bioconversion. Besides substrate, product and overlay tolerance, other factors such as the salt concentration and the presence of toxic lignin breakdown products could decrease the efficiency of the process. Therefore, the selection of an organism that can tolerate harsh media conditions and the development of appropriate feeding strategies are critical factors in lignin-derived bioprocesses. The approach described here resulted in an almost complete extraction of the product in the organic phase and higher 4VP concentrations than previously reported when using biological catalysts to produce 4VP from pure CA [8, 10, 16, 17] or lignocellulosic biomass [25].

## Conclusions

This work compared the performance of three organisms in a 4VP production process and demonstrates its production from CA extracted from a solid lignin-rich corn stover bioprocess residue. We found that the plasmid expression of a PAD enzyme in *C. glutamicum* strains resulted in the highest 4VP concentrations reported to date in any culture medium. In contrast, *B. subtilis* showed the lowest performance of the recombinant strains in all tested conditions. Continuous extraction of 4VP from the liquid phase with an organic overlay enabled high product concentrations and yields in batch mode, and the best results (187 g/L of 4VP from pure CA in the overlay with a 90% yield) were obtained when using undecanol. The use of undecanol also has the added benefit of enabling recovery of pure 4VP via a simple vacuum distillation due to the > 40 ℃ difference in boiling points between 4VP (189–206 ℃) and undecanol (243 ℃). Our results showcase *E. coli* as the top performing organism in the lignin liquors, *C. glutamicum* as a promising new host for the high-level production of 4VP, and also indicate that the implementation of an extractive fermentation process could be beneficial to increase the conversion and recovery at higher scales. Overall, the combination of 4VP conversion host and extractive overlay described in his study outline a process that has a clear route to industrialization for this bioplastic monomer.

## Methods

### Chemicals and media

*p*-coumaric acid, dodecane, octanol, decanol, undecanol, diethyl ether, and all components of M9 medium were purchased from Sigma Aldrich. A 10% 4-vinylphenol solution in propylene glycol was purchased from Alfa Aesar. Tryptic Soy Broth was purchased from BD Difco and prepared according to the manufacturer’s instructions. Coumaric acid (98%) powder was added to tryptic soy broth at 50 ºC and solubilized by the slow addition of 10 N NaOH until the pH was approximately 7.5, then the medium was filter-sterilized using a 0.45 µm surfactant-free cellulose acetate membrane. M9 medium contained the following components (per liter): 6.78 g of Na_2_HPO_4_, 3 g of KH_2_PO_4_, 0.5 g of NaCl, 1.98 g of (NH_4_)SO_4_, 240 mg of MgSO_4_, 12 mg of CaCl_2_, 160 µg of CuSO_4_, 160 µg of MnSO4, 160 µg of FeSO4, 160 µg of ZnSO4, 20 mg of thiamine, and 20 mg of biotin. A tenfold concentrated M9 solution was used to supplement lignin liquors in a 1:9 v/v ratio to be used as cultivation media for production experiments.

*E. coli* and *B. subtilis* were maintained on LB (10 g/L tryptone, 5 g/L yeast extract, and 5 g/L NaCl) plates or liquid and *C. glutamicum* was maintained on LB agar/liquid or LBHIS agar plates (5 g/L tryptone, 5 g/L NaCl, 18.5 g/L brain heart infusion, 15 g agar, and 91 g/L sorbitol). *C. glutamicum* electroporation media are as follows; electrocompetent medium-1: autoclave 1 g tryptone, 0.5 g yeast extract, 1 g NaCl, 2.5 g LB media in 80 mL water and add filter sterilized 2.5 g glycine and 50 µL tween80, in 20 mL water; and BHIS: 18.5 g/L brain heart infusion and 91 g/L sorbitol [13].

### Plasmid construction

The gene coding for a phenolic acid decarboxylase (PAD) enzyme in *B. amyloliquefaciens* was codon-optimized for *B. subtilis*, *C. glutamicum*, and *E. coli*, and synthesized by Genscript (Piscataway, NJ, USA). The *B. subtilis* PAD gene was then cloned into plasmid pHT08 to generate plasmid pJAM90 (Cm^R^) via Gibson assembly using the DIVA platform at the Joint BioEnergy Institute. The codon-optimized genes for *E. coli* and *C. glutamicum* were cloned into the shuttle plasmid pZ8-Ptac to generate plasmids pJAM89 (Km^R^) and pJAM88 (Km^R^), respectively. All vectors allowed for the IPTG-inducible expression of the PAD enzyme.

To generate a clean deletion of *phdA* in *C. glutamicum* a deletion construct in a *sacB* suicide vector was made. The upstream and downstream regions of *phdA* (Cgl0284) were PCR amplified using *C. glutamicum* wild type genomic DNA with the following primers (sequences are shown in parentheses): KOphdA-F1-Eco (GTCgaattcCGATGACAGATGCACCCTGA), KOphdA-R1 (actagtgttcagtcgatcgttcgaaAGATCACGTTTGAAGTCA), KOphdA-F2 (ttcgaacgatcgactgaacactagtCACTTGGAGAACGCAATGA), and KOphdA-R2-HindIII (ATGaagcttGCGTCCGAAGAGGTCGGATA). The splice overlap PCR product, made by amplifying the upstream and downstream PCR product with primers KOphdA-F1-Eco and KOphdA-R2-HindIII, was ligated into zero blunt vector (Invitrogen). It was subsequently cut with EcoRI and HindIII and ligated into the similarly cut pK18mobsacB. The strains and plasmids used in this study are listed in Table 1 and those generated in this work can be accessed at https://public-registry.jbei.org using the part ID numbers provided.

### Generation of strains

The microorganisms used in this work are described in Table 1. A *C. glutamicum* ATCC 13,032 strain that is unable to metabolize coumaric acid was generated by making a clean deletion of *phdA,* the gene coding for an acyl-CoA ligase (Cgl0284). This was accomplished by performing double homologous recombination and selection via the *sacB* system [9]. To integrate the deletion construct into *C. glutamicum* via transformation*,* wild type cells were made electrocompetent as per van der Rest with some variations [22]. *C. glutamicum* grown on LBHIS plates was inoculated into 10 mL LB 2% glucose and grown overnight at 200 rpm and 30 ℃. The overnight culture was inoculated into 1 L of electrocompetent medium-1 to an OD600nm of 0.3 and grown at 200 rpm at 18 ℃ until an OD600nm of 0.9–1.0. The cells were harvested, washed four times with ice cold 10% v/v glycerol, resuspended in 1 mL 10% v/v glycerol, and 150 μL aliquots were flash frozen in liquid nitrogen and stored at -80 ℃ until time of electroporation. The transformation to introduce the *pdhA* deletion construct was done by thawing the electrocompetent cells on ice and electroporating the cells in a 0.2 mm electroporation cuvette with 1 μg of plasmid using the conditions of 25 μF, 200 Ω, 3 kV, for 5 ms. Cells were resuspended in prewarmed (45 °C) 940 μL BHIS and incubated for 9 min at 45 °C. The cells recovered at 200 rpm at 30 ℃ for 5 h before plating cells onto LBHIS agar supplemented with 30 μg/mL kanamycin and incubated at 30 °C. The mutants were verified by PCR and by the inability to grow in CA as the sole carbon source.

In this study, strains with expression plasmids were generated by electroporation and briefly described here. *E. coli* DH5α Mix & Go competent cells were purchased from Zymo Research and transformed by incubation with 1 µg of the plasmid DNA pJAM88 at room temperature for 5 min before plating on LB medium plates containing 30 µg/mL of kanamycin. *B. subtilis* RIK1285 cells were purchased from Takara Bio and made competent by harvesting them at the onset of stationary phase, following the instructions described by the supplier. Fresh competent cells were transformed with 2 µg of plasmid DNA pJAM90 and plated on LB plates with 10 µg/mL of chloramphenicol. *C. glutamicum* wild-type and the *ΔphdA* mutant were transformed by electroporation as above with these modifications. Both strains were made electrocompetent, thawed, and gently mixed with 100–200 ng of plasmid DNA (pJAM89), electroporated and allowed to recover for 2 h at 200 rpm at 30 ℃ before plating onto LBHIS with 30 μg/mL kanamycin. Transformants were verified by PCR and enzymatic digestion of the extracted plasmids.

### Microbial cultivations

Cultivations were performed in 48-well flat bottom clear plates (Corning, USA) when an organic overlay was not added, in 48-well FlowerPlates (m2p, Germany) when an overlay was added to rich medium, and in 14 mL round-bottom plastic tubes for lignin liquors with an overlay. Seed cultures of all strains listed in Table 1 were started by inoculating cells from agar plates into tubes with 5 mL of tryptic soy broth and incubating at 30 ºC with vigorous shaking for 12 h. For the experiments in the clear plates, inocula were prepared by mixing 1 mL of the seed cultures with 9 mL of fresh tryptic soy broth and incubated for 8 h. IPTG (0.5 mM final concentration) was added to the inocula after 6 h of incubation. Kanamycin (30 µg/mL) or chloramphenicol (10 µg/mL) were added as required for plasmid maintenance. To start the experiments in the clear plates, 15 µL of the inocula were combined with 485 µL of tryptic soy broth, with or without supplementation of 4VP or CA, and incubated with the plastic lid on in a shaking platform at 30 ºC and 200 rpm. The optical density at 595 nm was measured using a DTX880 plate reader (Beckton-Coulter, USA).

For the experiments in the FlowerPlates or culture tubes, seed cultures were started in 5 mL of tryptic soy broth and incubating at 30 ºC with vigorous shaking for 12 h with the appropriate antibiotics at the concentrations described above. Cells were transferred to 250 mL flasks containing 50 mL of tryptic soy broth (at a 1/10 dilution) and incubated at 30 ºC with shaking in the presence of antibiotics. IPTG (0.5 mM final concentration) was added to the flasks after 6 h and incubated again for 2 h. Cell densities were measured, and the cells were centrifuged and resuspended in tryptic soy broth with CA or lignin liquors to obtain an initial OD of 4. IPTG (0.5 mM final concentration) was added at the beginning of the cultivations with plasmid-harboring strains. For cultivations in FlowerPlates, 800 µL of cells in tryptic soy broth media were combined with 200 µL of octanol, decanol or undecanol, covered with a gas-permeable sealing foil with a reduced evaporation layer (m2p, Germany), and incubated inside a humidity-controlled incubator shaker set at 30 ºC and 900 rpm. After 48 h, the entire contents of each Flowerplate well were collected in 1.5 mL tubes and the dodecane layer, supernatant, and cells were separated by centrifugation. For cultivations in culture tubes, 3.2 mL of cells in lignin liquors were combined with 0.8 mL of undecanol and incubated at 30 ºC and 200 rpm. Samples from the aqueous phase and undecanol phase were collected every 12 h. The cells in the aqueous phase were briefly centrifuged, resuspended in water, and transferred to a 96-well Costar black clear bottom plates (Corning, USA) to measure OD 600 nm using a SpectraMax m2 (Molecular Devices, USA). Each fraction was kept at − 20 ºC until analysis. All cultivations were performed in triplicate.

### Analytical methods

CA and 4VP were quantified by HPLC in samples taken from the aqueous and organic phases of the cultivations or lignin liquors after diluting with water (aqueous samples) or pure acetonitrile (organic samples). The analysis was performed with an Agilent Technologies 1200 series instrument equipped with an Eclipse Plus Phenyl-hexyl column (250 mm length, 4.6 mm diameter, 5 µm particle size; Agilent Technologies, USA) kept at 50 ºC, and using an injection volume of 5 µl. The mobile phase was composed of 10 mM ammonium acetate in water (solvent A) and 10 mM ammonium acetate in acetonitrile 90% (solvent B), prepared from a stock solution of 100 mM ammonium acetate and 0.7% formic acid in water. The following mobile phase gradient profile was used: 30% B (0 min; 0.5 mL/min), 80% B (12 min; 0.5 mL/min), 100% B (12.1 min; 0.5 mL/min), 100% B (12.6 min; 1 mL/min), 30% B (12.8 min; 1 mL/min), 30% B (15.6 min; 1 mL/min). A Poroshell 120 EC-C18 column (50 mm length, 3 mm diameter, 2.7 µm particle size; Agilent Technologies, USA) kept at 22 ºC was also employed for high-throughput analysis of samples collected from FlowerPlate cultivations, by using 40% (v/v) acetonitrile in water and 0.04% formic acid as mobile phase. Coumaric acid and 4-vinylphenol were quantified with a UV detector using 310 nm and 254 nm wavelengths, respectively. Analyte concentrations were calculated by comparison of the resulting peak areas to calibration curves made with commercial compounds.

### Lignin preparation, depolymerization and coumaric acid extraction

Corn stover was pretreated in a horizontal screw reactor at 175 ºC with 30 g H_2_SO_4_/kg dry biomass, 30% (w/w) total solids loading, and a residence time of 8 min (Shekiro III). The pretreated corn stover was then neutralized with 40% (w/w) NaOH to pH 5.5 before loading of the enzymes. Enzymatic saccharification was performed in a 50 L IKA SPP50 reactor with 15% (w/w) solid loading. The total batch mass was 30 kg with cellulose (CTec2) 64 mg protein/g dry biomass and xylanase (HTec2) 8 mg protein/g dry biomass. After 96 h incubation, the reaction was stopped, and the slurry was transferred out. Lignin rich solid was separated from the hydrolysate using a basket centrifuge with a polypropylene filter bag (pore size 25–30 micron) as the liner.

The lignin-rich residue obtained after saccharification was washed with water to remove low molecular weight compounds. This material was oven-dried at 50 ºC for 48 h, ground with a mortar and pestle and milled using a 2 mm sieve. Compositional analysis of the dried sample revealed lignin, glucan, and xylan contents of 54.5%, 13.7%, and 3.8%, respectively. This sample was subjected to a base-catalyzed depolymerization process, as follows: (1) 37.5 g of solids were mixed with 150 mL of 5% NaOH in a stainless steel pressure reactor containing an impeller and a jacket for temperature control (Buchiglas, Switzerland); (2) a reaction was carried at 120 ºC and for 30 min (in addition to a 35 min heating up and a 25 min heating down ramps); (3) a lignin liquor with a pH of 12 was recovered and the pH was adjusted to 7.5 with concentrated H_2_SO_4_; (4) the liquid phase was recovered by centrifugation and filtered through 0.45 µm surfactant-free cellulose acetate membranes. The recovered liquor was supplemented with M9 salts (1/10 vol. of a 10 × M9 stock), resulting in a concentration of 8 g/L of CA.

## Supplementary Information

**Additional file 1.** HPLC–UV chromatogram obtained from the lignin liquor at pH 7.5 prepared in this work and used for bioconversion. The sample was diluted 100-fold in water before analysis.

## Data Availability

All data generated and analyzed during this study are included in this article and its additional files.
