# Supplementary figures and images for: Evaluation of bacterial hosts for conversion of lignin-derived p-coumaric acid to 4-vinylphenol
Source: Microb Cell Fact. 2021 Sep 15;20:181. doi: 10.1186/s12934-021-01670-8 (PMC8442356; doi:10.1186/s12934-021-01670-8)

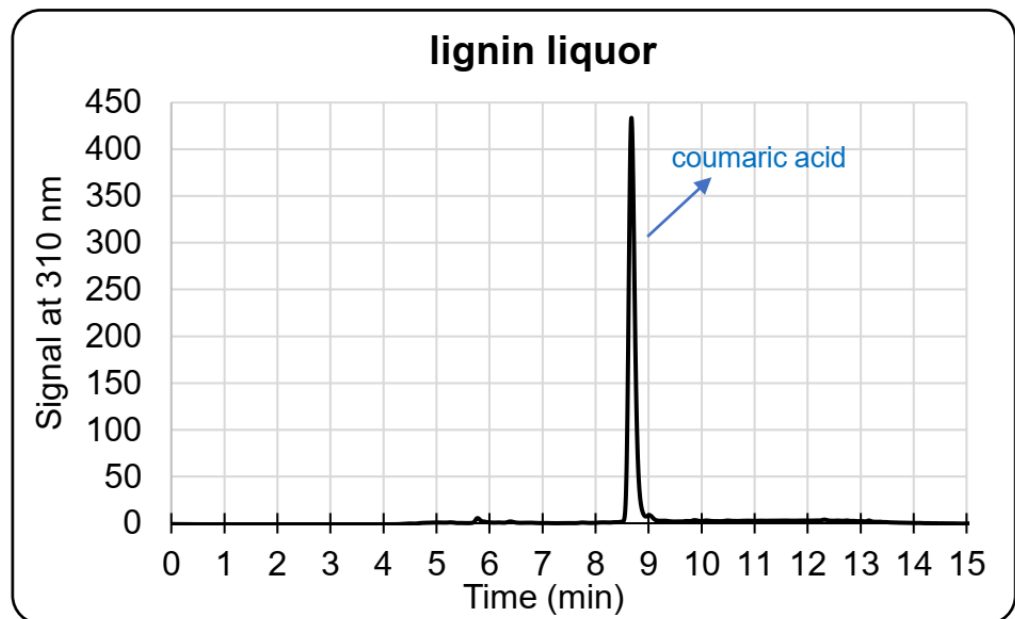

Additional file 1

Supplement: Supplementary file 1 — Additional file 1. HPLC–UV chromatogram obtained from the lignin liquor at pH 7.5 prepared in this work and used for bioconversion. The sample was diluted 100-fold in water before analysis. [file 12934_2021_1670_MOESM1_ESM.pdf]
